# Galectin-3 mediates bone marrow microenvironment-induced drug resistance in acute leukemia cells via Wnt/β-catenin signaling pathway

**DOI:** 10.1186/s13045-014-0099-8

**Published:** 2015-01-27

**Authors:** Kaimin Hu, Yanjun Gu, Lixia Lou, Lizhen Liu, Yongxian Hu, Binsheng Wang, Yi Luo, Jimin Shi, Xiaohong Yu, He Huang

**Affiliations:** Bone Marrow Transplantation Center, The First Affiliated Hospital, School of Medicine, Zhejiang University, Hangzhou, 310003 China; Cancer Institute, The Second Affiliated Hospital, School of Medicine, Zhejiang University, Hangzhou, 310003 China; Department of Surgical Oncology, The First Affiliated Hospital, School of Medicine, Zhejiang University, Hangzhou, 310003 China

**Keywords:** Galectin-3, Acute leukemia, Bone marrow mesenchymal stromal cell (BM-MSC), β-catenin, Drug resistance

## Abstract

**Background:**

Acute leukemia is currently the major cause of death in hematological malignancies. Despite the rapid development of new therapies, minimal residual disease (MRD) continues to occur and leads to poor outcomes. The leukemia niche in the bone marrow microenvironment (BMM) is thought to be responsible for such MRD development, which can lead to leukemia drug resistance and disease relapse. Consequently further investigation into the way in which the leukemia niche interacts with acute leukemia cells (ALCs) and development of strategies to block the underlying process are expected to improve disease prognosis. Recent studies indicated that galectin-3 (gal-3) might play a pivotal role in this process. Thus we aimed to elucidate the exact role played by gal-3 in this process and clarify its mechanism of action.

**Methods:**

We used human bone marrow-derived mesenchymal stromal cells (hBM-MSCs) to mimic the leukemia BMM *in vitro*, and investigated their effects on drug resistance of ALCs and the possible mechanisms involved, with particular emphasis on the role of gal-3.

**Results:**

In our study, we demonstrated that hBM-MSCs induced gal-3 up-regulation, promoting β-catenin stabilization and thus activating the Wnt/β-catenin signaling pathway in ALCs, which is critical in cytotoxic drug resistance of leukemia. This effect could be reversed by addition of gal-3 short hairpin RNA (shRNA). We also found that up-regulation of gal-3 promoted Akt and glycogen synthase kinase (GSK)-3β phosphorylation, thought to constitute a cross-bridge between gal-3 and Wnt signaling.

**Conclusions:**

Our results suggest that gal-3, a key factor mediating BMM-induced drug resistance, could be a novel therapeutic target in acute leukemia.

## Background

Acute leukemia (AL), mainly consisting of acute myeloid leukemia (AML) and acute lymphoblastic leukemia (ALL), is currently the major cause of death in hematological malignancies, affecting patients of all ages. Despite ongoing improvements in the outcomes of patients with AL, only 30%-40% of adult ALL patients achieve long-term, disease-free survival due to drug resistance and disease relapse [1,2]. AML is a heterogeneous disease, and a substantial number of AML patients have quite a low cure rate even after hematopoietic stem cell transplantation [3,4]. Minimal residual disease (MRD), which is widely considered an independent prognostic factor and currently attracts much attention in treatment intervention, has become a vital challenge in the search for a cure for AL [5-7]. In addition, the leukemia niche is believed to play a critical role in the development of MRD.

The leukemia niche, composed of the osteoblastic and vascular bone marrow niche, provides a home for malignant cells and is responsible for disease relapse as well as treatment resistance. Previous studies have shown that stromal cells in the bone marrow microenvironment (BMM) play an important role in leukemia genesis and progress by secreting various chemicals and contacting signals [8], for example the axis of VCAM-1/VLA-4 [9], SDF-1/CXCR4 [10], and Notch [11], as shown *in vitro* and *in vivo*. Since mesenchymal stromal cells (MSCs), an important component of both the solid and hematologic tumor microenvironment [12], give rise to different stromal cell lineages [13]. In our study we used human bone marrow-derived MSCs (hBM-MSCs) to represent a relatively homogeneous BMM stromal cell population with hematopoiesis-supporting capabilities and immune-regulatory properties.

Galectin-3 (gal-3), a 30-kDa protein without enzymatic activity, is a member of the β-galactoside-specific lectin family. Gal-3 exhibits pleiotropic biological functions especially in tumors. It has roles in cell growth, apoptosis, adhesion, tumor angiogenesis, malignant cell metastasis, cancer-matrix interaction and also cancer drug resistance [14,15]. Recent evidence revealed that gal-3 was up-regulated in Ph^+^ chronic myeloid leukemia (CML) and in pre-B ALL after conditioning with BM stromal cells [16,17]. Cheng and colleagues [18] reported that in patients with AML, higher bone marrow *LGALS3*(*gal*-*3*) gene expression was an independent unfavorable prognostic factor for overall survival.

However, the specific role of gal-3 in BMM-induced drug resistance of acute leukemia cells (ALCs) has not yet been investigated. The aim of our study was to identify the specific mechanism involved. We found that gal-3 was dramatically up-regulated in hBM-MSC-conditioned AL cell lines, accompanying activation of β-catenin signaling. Both gal-3 and β-catenin signaling were essential in promoting the survival of ALCs when treated with cytotoxic drugs. We also showed, for the first time, that gal-3 modulated β-catenin signaling by regulating GSK-3β phosphorylation and the PI3K/Akt pathway in hBM-MSC-conditioned ALCs.

## Results

### hBM-MSCs induce gal-3 expression and drug resistance in ALCs

**Figure 1:**
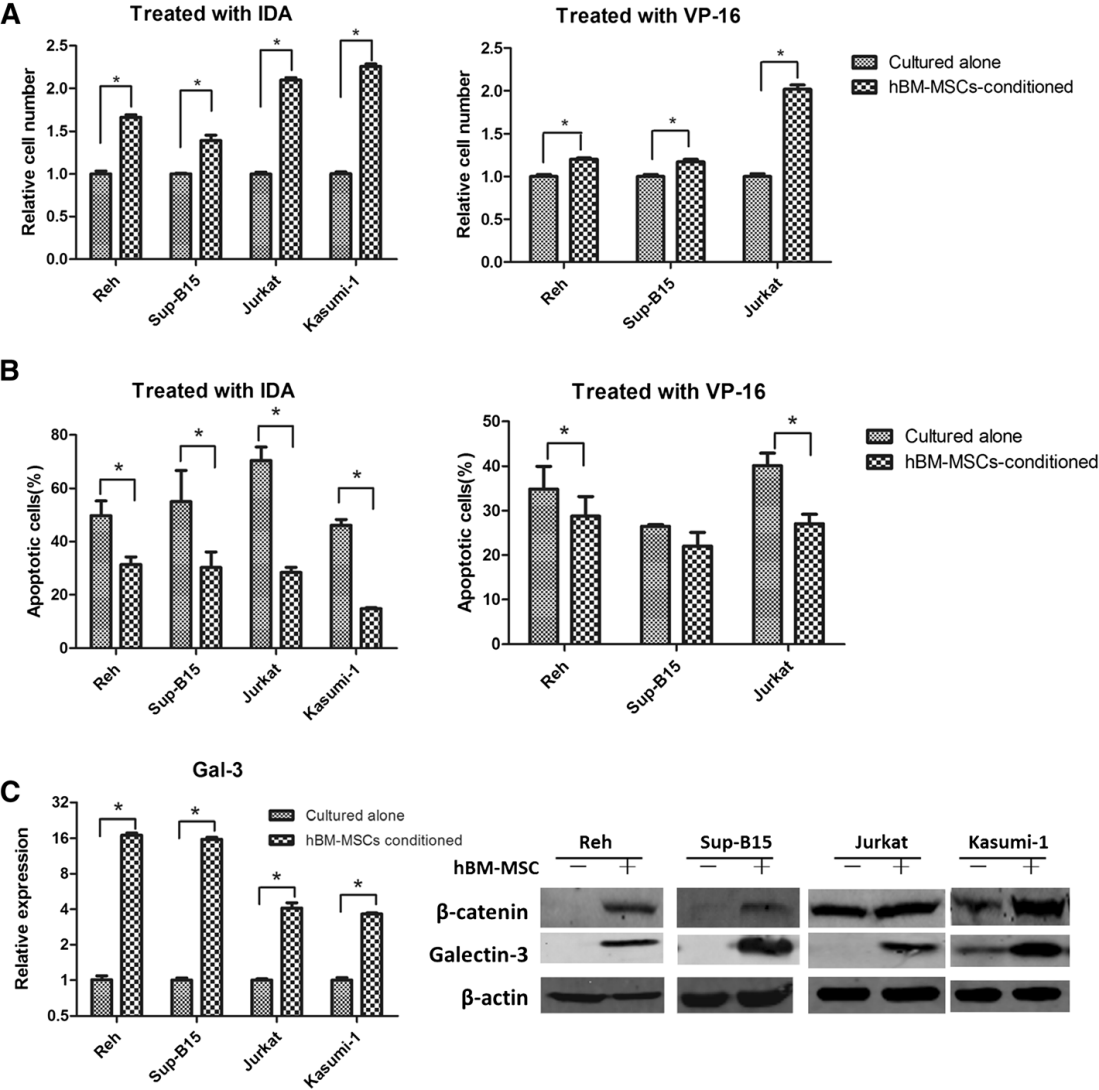
**hBM-MSCs protect ALCs against cytotoxic drugs by up-regulation of gal-3 at both the mRNA and protein levels. (A)** ALCs were treated with IDA (25 nM for Reh and Jurkat cells, 20 nM for Sup-B15, 15 nM for Kasumi-1) or VP-16 (250 nM for Reh cells, 200 nM for Sup-B15, 1000 nM for Jurkat), cultured with or without hBM-MSCs. The relative number of surviving ALCs was analyzed with CCK-8 assay, as indicated. Absorbance of ALCs cultured alone were defined as 1. **(B)** The apoptotic level of ALCs treated with IDA or VP-16, cultured with or without hBM-MSCs was determined by FACS. **(C)** ALCs cultured with hBM-MSCs expressed higher level of gal-3 and β-catenin, compared with those unconditioned.

**Figure 2:**
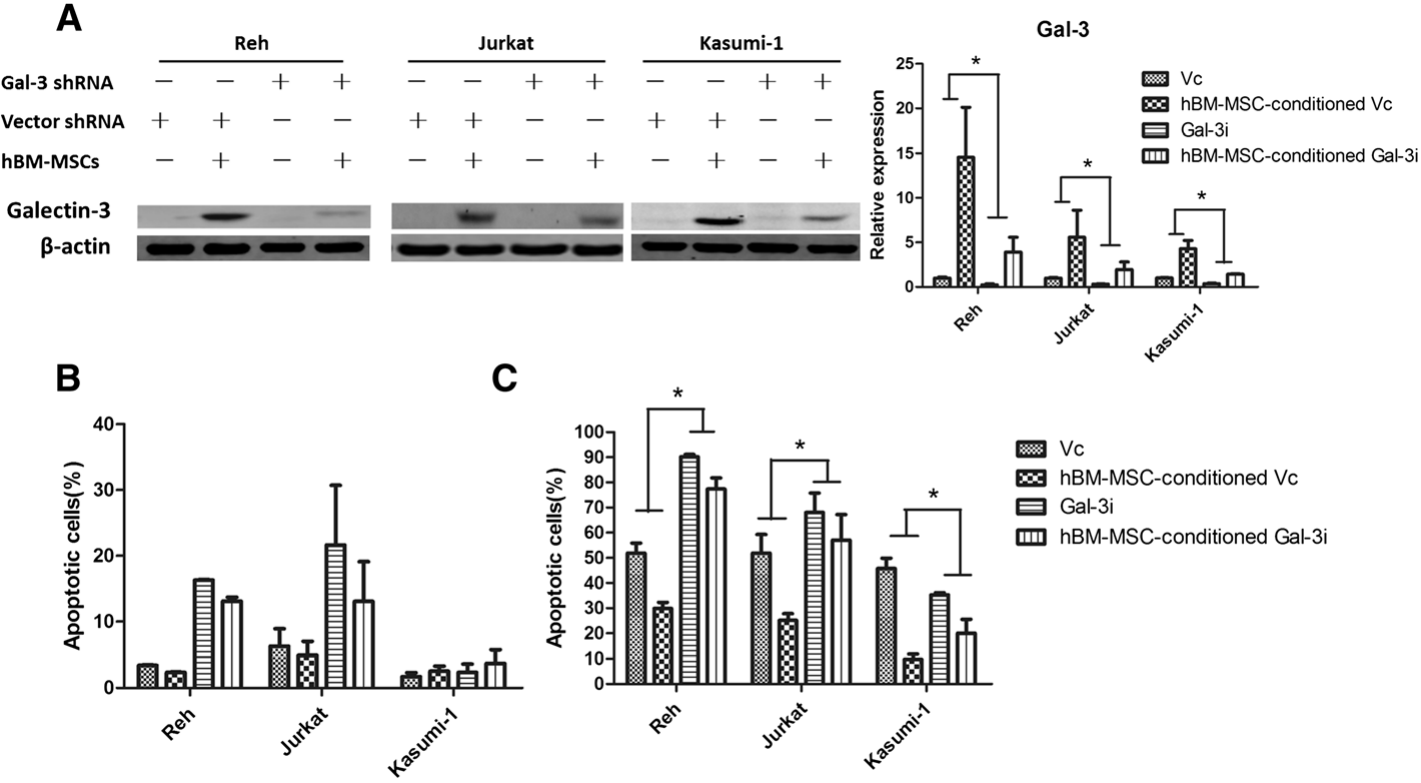
**Gal-3 is necessary for drug resistance of ALCs induced by hBM-MSCs. (A)** ALCs (Reh, Jurkat and Kasumi-1 cells) were transfected by gal-3 shRNA or vector as control. Western blot and real time PCR analysis of gal-3 in transfected ALCs under different conditions, namely cultured alone or with hBM-MSCs. **(B)** The apoptotic levels of transfected ALCs without any cytotoxic drugs. **(C)** The apoptotic levels of transfected ALCs treated with IDA at the concentrations described above. Vc: cells transfected with vector; Gal-3i: cells transfected with gal-3 shRNA.

Gal-3 was recently reported to be associated with the promotion of drug resistance in CML by the bone marrow microenvironment [16]. We therefore examined whether it also applied to ALCs. We used hBM-MSCs to mimic the leukemia BMM *in vitro* and validated the ability of hBM-MSCs to protect ALCs from cytotoxic drugs such as IDA and VP-16. Our results showed that when ALCs were exposed to cytotoxic drugs, hBM-MSCs significantly augmented the absolute number of surviving cells. Apoptotic levels of ALCs were also significantly decreased when co-cultured with hBM-MSCs (Figure 1A and B, *P* < 0.05). We also examined the expression level of gal-3 and found that both mRNA and protein level were up-regulated in all four AL cell lines conditioned by hBM-MSCs (Figure 1C). To further elucidate the exact role of hBM-MSC-induced gal-3 up-regulation in drug resistance of ALCs, we silenced gal-3 in Reh, Jurkat and Kasumi-1 cells by stable transfection of gal-3 antisense shRNA. The results showed that gal-3 was knocked down by more than 60% in all three cell lines (Figure 2A). We found that the apoptotic levels in gal-3-silenced ALCs increased significantly with or without IDA, especially in Jurkat and Reh cells, and the protective effects of hBM-MSCs against IDA were also weakened (Figure 2B and C). These findings suggest that gal-3 induced by hBM-MSCs at least partially explained drug resistance of acute leukemia cells *in vitro*.

### Up-regulation of gal-3 promotes β-catenin stabilization

**Figure 3:**
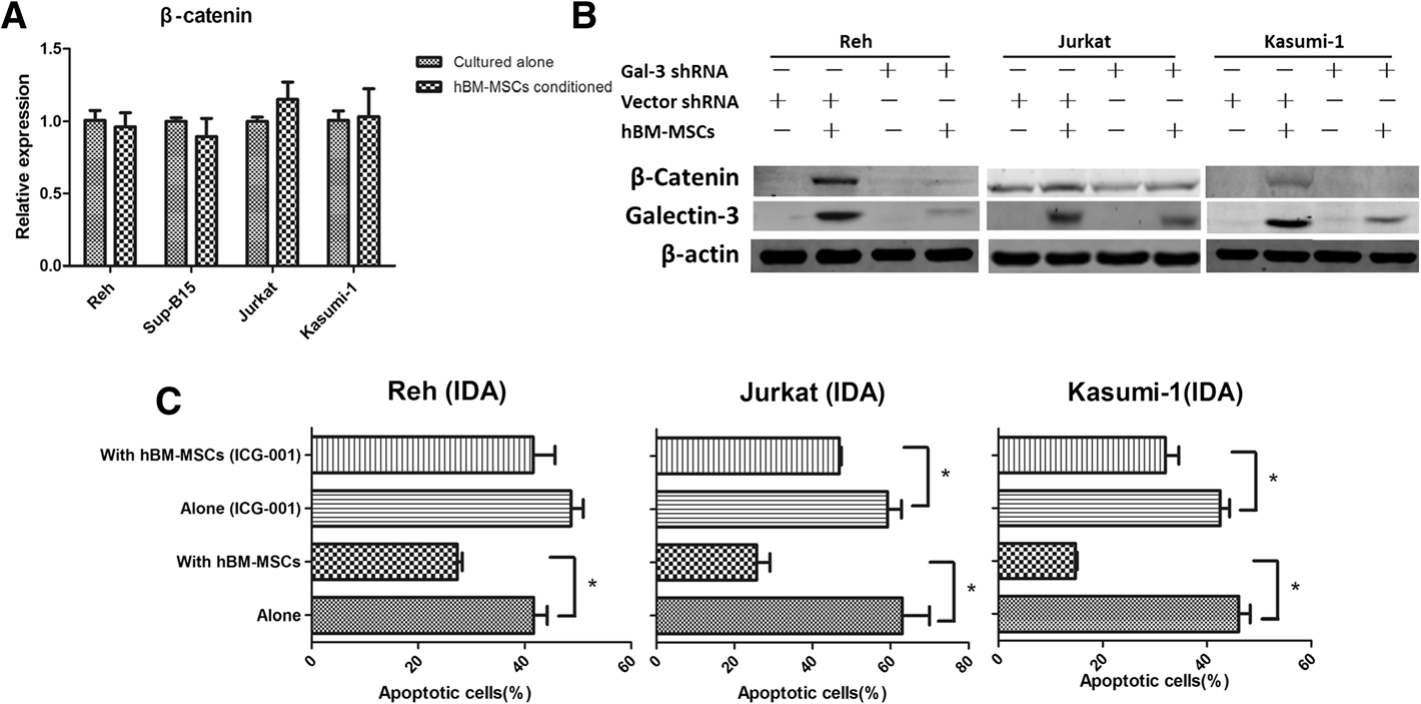
**hBM-MSC-induced β-catenin stabilization is essential for the drug resistance of ALCs, and is eliminated by gal-3 knockdown. (A)** The transcriptional level of β-catenin in all four cell lines cultured alone or with hBM-MSCs. **(B)** The expression of β-catenin and gal-3 in ALCs after transfection with gal-3 shRNA or vector, cultured alone or with hBM-MSCs. **(C)** ALCs, cultured alone or with hBM-MSCs, were pretreated with ICG-001 (5 μM for Reh cells, 10 μM for Jurkat and Kasumi-1) for 30 minutes, then IDA at indicated concentration was added. Apoptosis was measured after exposure to IDA for 48 h.

Gal-3 has been indicated to play a vital role in Wnt signaling, which is associated with the promotion of cell cycle progression and cell viability in colon and pancreatic cancer cells by interacting with β-catenin [19,20]. Accordingly, we investigated whether this signaling pathway also played a role in hBM-MSC-conditioned ALCs. We first analyzed the expression levels of β-catenin, which showed that the protein level of β-catenin were dramatically higher after co-culture with hBM-MSCs, although the mRNA level did not show much difference (Figures 1C and 3A). This suggested that gal-3 might modulate β-catenin expression at the post-transcriptional level, perhaps by inhibiting its degradation. We then verified our hypothesis in gal-3-shRNA-transfected ALCs. Hardly any β-catenin up-regulation was observed when ALCs were co-cultured with hBM-MSCs (Figure 3B). To further investigate the role of gal-3-stabilized β-catenin in ALC drug resistance, we treated ALCs with ICG-001, a specific Wnt/β-catenin signaling inhibitor, and found that it dramatically decreased the protective effect of hBM-MSCs against the effects of IDA in ALCs (Figure 3C). Thus, this suggested that hBM-MSC-induced gal-3 promoted β-catenin stabilization, which was pivotal in drug resistance of ALCs.

### Gal-3 induces β-catenin accumulation and activates target gene expression of Wnt/β-catenin signaling

**Figure 4:**
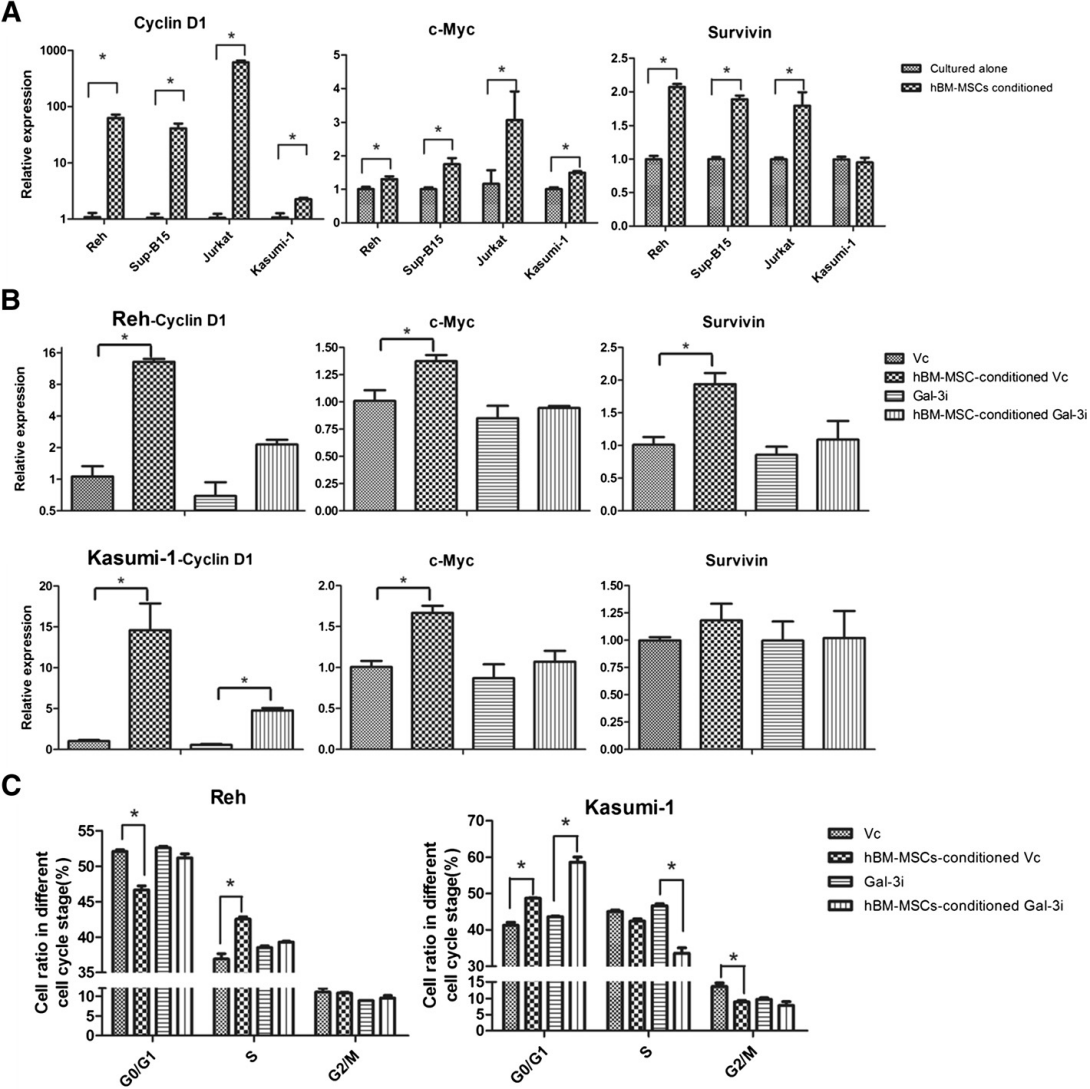
**Expression of target genes of Wnt/β-catenin signaling are up-regulated in hBM-MSC-conditioned ALCs. (A)** The mRNA levels of Cyclin D1, c-Myc and Survivin expressed in ALCs cultured alone or with hBM-MSCs. **(B)** The mRNA levels of Cyclin D1, c-Myc and Survivin expressed in Reh and Kasumi-1 cells after transfection with gal-3 shRNA or vector, cultured alone or with hBM-MSCs. **(C)** Cell cycle analysis of Reh and Kasumi-1 cells after transfection with gal-3 shRNA or vector, cultured alone or with hBM-MSCs.

We analyzed the target genes of the Wnt/β-catenin signaling pathway to verify that they were activated in our co-culture system. The results of qRT-PCR showed that transcription of cyclin D1, c-myc and survivin were all up-regulated in hBM-MSC-conditioned Reh cells, but that the up-regulation was much reduced in gal-3-silenced cells. Similar findings were also observed in Kasumi-1 cells (Figure 4A and B). In line with cyclin D1 expression, the proportion of proliferative cells increased in hBM-MSC-conditioned ALCs, and this effect was weakened once gal-3 was knocked down (Figure 4C). Collectively, these results showed that hBM-MSCs promoted activation of the Wnt/β-catenin signaling pathway in ALCs, which was mediated through gal-3.

### Gal-3 stabilizes β-catenin by regulating GSK-3β activity and PI3K/Akt axis

**Figure 5:**
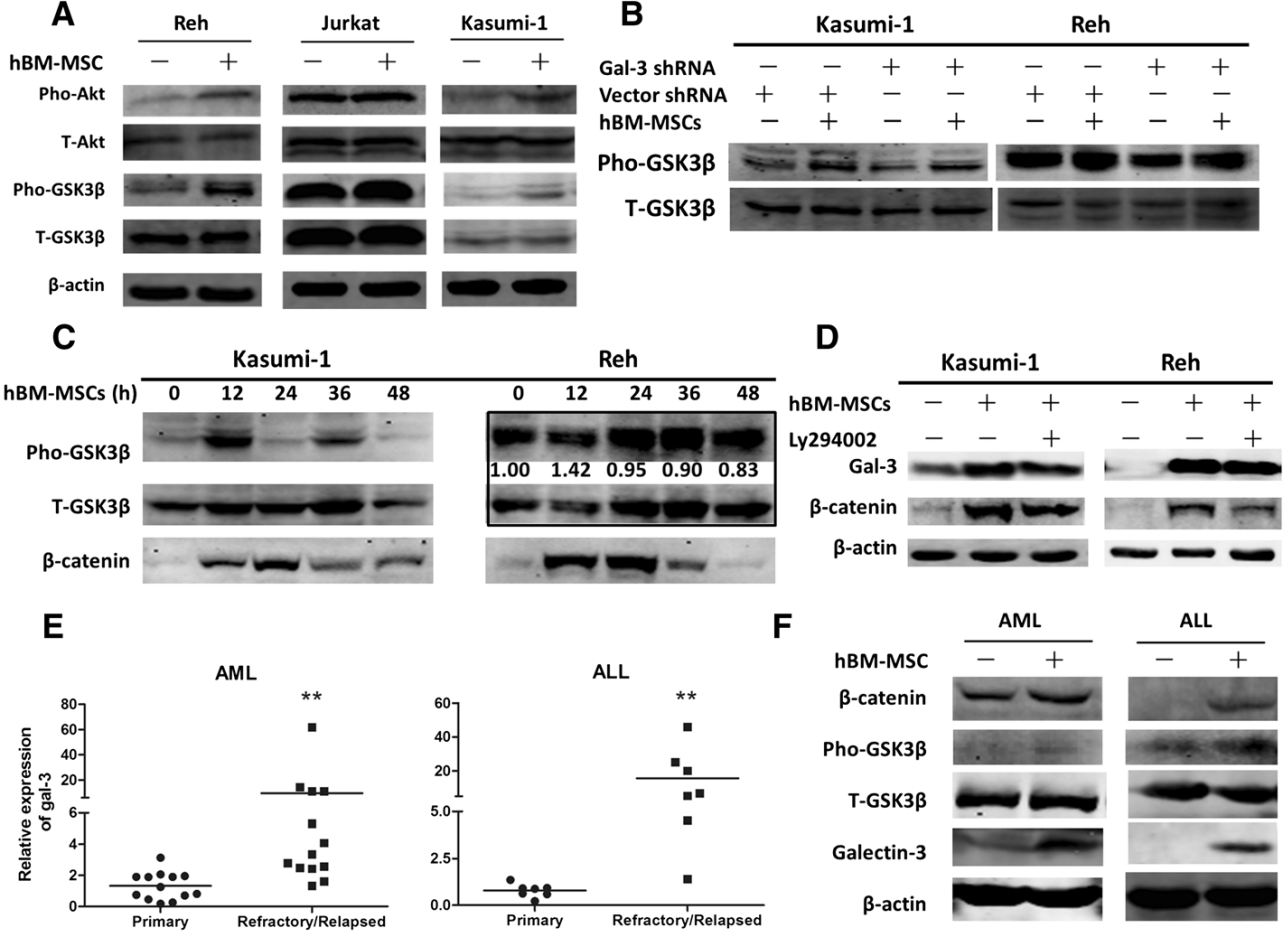
**Gal-3 up-regulation promotes phosphorylation of Akt and GSK-3β, thereby supporting β-catenin stabilization. (A)** The protein level of phosphorylated Akt (Pho-Akt), total Akt (T-Akt), phosphorylated GSK-3β (Pho-GSK-3β), and total GSK-3β (T-GSK-3β) expressed in ALCs cultured alone or with hBM-MSCs. Increased gal-3 in hBM-MSC-conditioned ALCs was associated with increased phosphorylation of Akt and GSK-3β while the total protein expression remained unchanged. **(B)** The protein level of Pho-GSK-3β and T-GSK-3β in gal-3-silenced Kasumi-1 and Reh cells, cultured alone or with hBM-MSCs. **(C)** The time-dependent changes of Pho-GSK-3β and β-catenin in hBM-MSC-conditioned Kasumi-1 and Reh cells. The densitometry data was measured using Quantity One software, calculated as Pho-GSK-3β/T-GSK-3β compared with the level at 0 h. **(D)** Expression of β-catenin after treatment with LY294002 (25 μM) in hBM-MSC-conditioned Kasumi-1 and Reh cells. **(E)** The mRNA levels of gal-3 expressed in AL patients (*P* < 0.01). We enrolled 40 patients in all, including 13 primary AML, 13 refractory/relapsed AML and 7 primary ALL, 7 refractory/relapsed ALL. **(F)** Western blot analysis of gal-3/β-catenin axis in AL patients-derived malignant cells cultured with or without hBM-MSCs. Data are from one AML and one ALL patients, representative of the patients examined.

Since gal-3 promoted β-catenin up-regulation at the protein but not the mRNA level, this suggested that it acted by modulating β-catenin at the post-transcriptional level, perhaps by inhibiting its degradation. To explore the specific mechanism involved, we assessed the expression of total and phosphorylated Akt and GSK-3β in hBM-MSC-conditioned ALCs, as previous studies have shown that PI3K/Akt signaling can promote GSK-3β phosphorylation which then stabilizes β-catenin [19,20]. Our results showed that increased gal-3 was associated with increased phosphorylation of GSK-3β, while the total protein expression remained unchanged (Figure 5A). In contrast in gal-3 silenced cells, the level of phosphorylated GSK-3β was not markedly changed even when the cells were conditioned with hBM-MSCs (Figure 5B). Our results also showed that up-regulation of β-catenin in ALCs occurred later than GSK-3β phosphorylation (Figure 5C), suggesting that gal-3 modulated β-catenin stabilization via GSK-3β. We also found increased phosphorylation of Akt in hBM-MSC-conditioned ALCs (Figure 5A). To confirm this hypothesis we treated the cells with LY294002, a specific PI3K/Akt signaling inhibitor, and found that the up-regulation of β-catenin mediated by hBM-MSCs was decreased (Figure 5D) while gal-3 expression was not significantly affected. Taken together, these results demonstrated that gal-3 reduced degradation of β-catenin by promoting phosphorylation of Akt and GSK-3β.

### Refractory/relapsed acute leukemia patients express higher level of gal-3 in bone marrow

Our research in different cell lines confirmed that gal-3 played an important role in hBM-MSC-induced drug resistance of ALCs *in vitro*, so we wondered whether it also applied to AL patients. Intriguingly, we found that gal-3 was expressed at a higher mRNA level in BM mononuclear cells from the patients who suffered from chemotherapy resistance or disease relapse both in AML and ALL, compared to primary AL patients (Figure 5E, *P* < 0.01). In addition, our results also confirmed that hBM-MSCs activated the gal-3/β-catenin signaling axis in primary malignant cells from AL patients (Figure 5F).

## Discussion

The present study demonstrates that gal-3 is specifically induced when acute leukemia cells (Reh, Sup-B15, Jurkat,Kasumi-1 and primary ALCs) are cultured with hBM-MSCs *in vitro*. Gal-3-shRNA largely eliminates hBM-MSC-induced gal-3 overexpression and reverses its protective effects against cytotoxic drugs in ALCs. Thus the induction of gal-3 is one of the pivotal underlying mechanisms of hBM-MSC-mediated protection of ALCs.

In view of the multiple biological functions of gal-3, we wondered how gal-3 performed its roles, especially in the leukemia BMM. Recent studies have suggested that gal-3 activates Wnt/β-catenin signaling in solid tumors, such as human colon and pancreatic cancer [19,20]. Wnt signaling plays an important role in maintaining normal hematopoiesis, and its degradation is causatively involved in the development of leukemia [21-23]. Yang *et al*. [24] indicated that Wnt signaling contributed to bone marrow stromal cell-mediated protection of ALL cells, which was in accordance with our results. However, the precise mechanisms involved remained unknown, especially in regard to the way in which gal-3 affected Wnt/β-catenin signaling between hBM-MSCs and acute leukemia. Our data were the first to reveal that gal-3 up-regulated β-catenin at the post-transcriptional level and activated its downstream signaling in the hBM-MSC-supported leukemia niche *in vitro*.

The specific known target genes of Wnt signaling include c-Myc [25], cyclin D1 [26], Survivin [27], gastrin [28], MMP-7 [29] and -2 [20], and cyclooxygenase-2 [30], most of which play an important role in cell survival, growth, self-renewal and motility. We therefore assessed the expression of these specific genes and detected increased transcription level of cyclin D1, survivin and c-Myc in ALCs conditioned by hBM-MSCs. However, once gal-3 was silenced, even though the cells were conditioned by hBM**-**MSCs, transcription of these genes was not significantly up-regulated. The increase in expression of cyclin D1 shown in our results was far greater than c-Myc and survivin. Lin *et al*. [31] found that gal-3 could promote cyclin D1 expression by enhancing its promoter activity through SP1 and a cAMP-responsive element in human breast epithelial cells. It is still unclear whether this also applies to acute leukemia cells. Further study will be necessary to confirm whether there are other mechanisms involved in gal-3-mediated cyclin D1 expression other than Wnt signaling in the acute leukemia microenvironment.

Our results illustrated that gal-3 activated target genes of Wnt/β-catenin pathway (Cyclin D1, c-Myc and Survivin). However, gal-3 inhibition in ALC itself did not significantly affect the expression of these genes. This might be due to the fact that ALCs only expressed limited gal-3 spontaneously. As previous studies have reported, Wnt/β-catenin pathway was required for leukaemogenesis [32] and a high frequency of leukemic cell lines were able to freely translocate cytosolic β-catenin to nucleus [33]. So we suggest that gal-3 is not the predominant regulator of Wnt/β-catenin pathway in ALCs without stimulation of hBM-MSCs.

We also analyzed the expression of pho-Akt and pho-GSK-3β in ALCs conditioned by hBM-MSCs, and found that both pho-Akt and pho-GSK-3β increased before the accumulation of β-catenin, which was consistent with previous studies showing that PI3K-activated Akt can phosphorylate GSK-3β at Ser^9^, thereby inactivating GSK-3β and triggering related signaling pathways [34-36]. In addition, Song [19], Kobayashi [20] and their colleagues reported that PI3K/Akt-inactivated GSK-3β might be the bridge between gal-3 and Wnt signaling in colon and pancreatic cancer. Taken together with our results, these findings suggest that Akt phosphorylation may be the first step that occurs after hBM-MSC-induced gal-3 up-regulation in ALCs, which subsequently promotes GSK-3β phosphorylation and supports β-catenin stabilization. Interestingly, the phosphorylation levels of β-catenin fluctuated behind GSK-3β after a time-lag, which indicates there may be a feedback loop between pho-GSK-3β and β-catenin stabilization.

Our results demonstrate that signals regulated by gal-3 are correlated with cell cycle progression and drug resistance, which may be major effects of leukemia microenvironments. This suggests the possibility that gal-3 could be a potential treatment target for acute leukemia, especially for minimal residual disease maintained by the leukemia niche. GCS-100, a citrus pectin-derived specific gal-3 antagonist, has proven to be effective in restoring or augmenting drug sensitivity in myeloma, large B-cell lymphoma and B-chronic lymphocytic leukemia [37-40]. Thus GCS-100, targeting the bone marrow microenvironment of acute leukemia, may be an innovative therapeutic molecule in the near future. Further *in vivo* researches and clinical trials are still warranted.

Since gal-3 is expressed in many tissues and cells, we tested whether hBM-MSCs also expressed gal-3, and found that they did (data not shown). Liu *et al*. [41] showed that human umbilical cord MSCs expressed gal-3 both on the cell surface as well as in secreted form. Secreted gal-3 was critical for the immunomodulatory potency of hUC-MSCs. It was also mentioned that OP9 cells also secreted gal-3 and that some of the gal-3 detected on pre-B ALL cells was of stromal origin [17]. The role of gal-3 derived from stromal cells and the potential mechanisms involved in its action in leukemia therefore deserve further study.

Yamamoto, *et al.* [16] have reported gal-3 is predominantly expressed in CML cells, but not in acute leukemias. Our results showed that hBM-MSCs also induced the up-regulation of gal-3 in ALCs *in vitro*. Furthermore, the expression level of gal-3 in refractory/relapsed AL patients is predominantly higher than that in primary ones. This may suggest gal-3 play a pivotal role in the maintance of MRDs and development of drug resistance. However, mechanisms about how the leukemic niche modulates expression of gal-3 remain little understood, since no mutation of the *LGALS3* gene has been detected. Since epigenetic alterations are important in development and maintenance of leukemia cells [42], it is still unknown whether gal-3 is promoted by activation of its transcription factors, DNA methylation, histone modifications, or action of non-coding RNAs which target gal-3 [43].

## Conclusions

**Figure 6:**
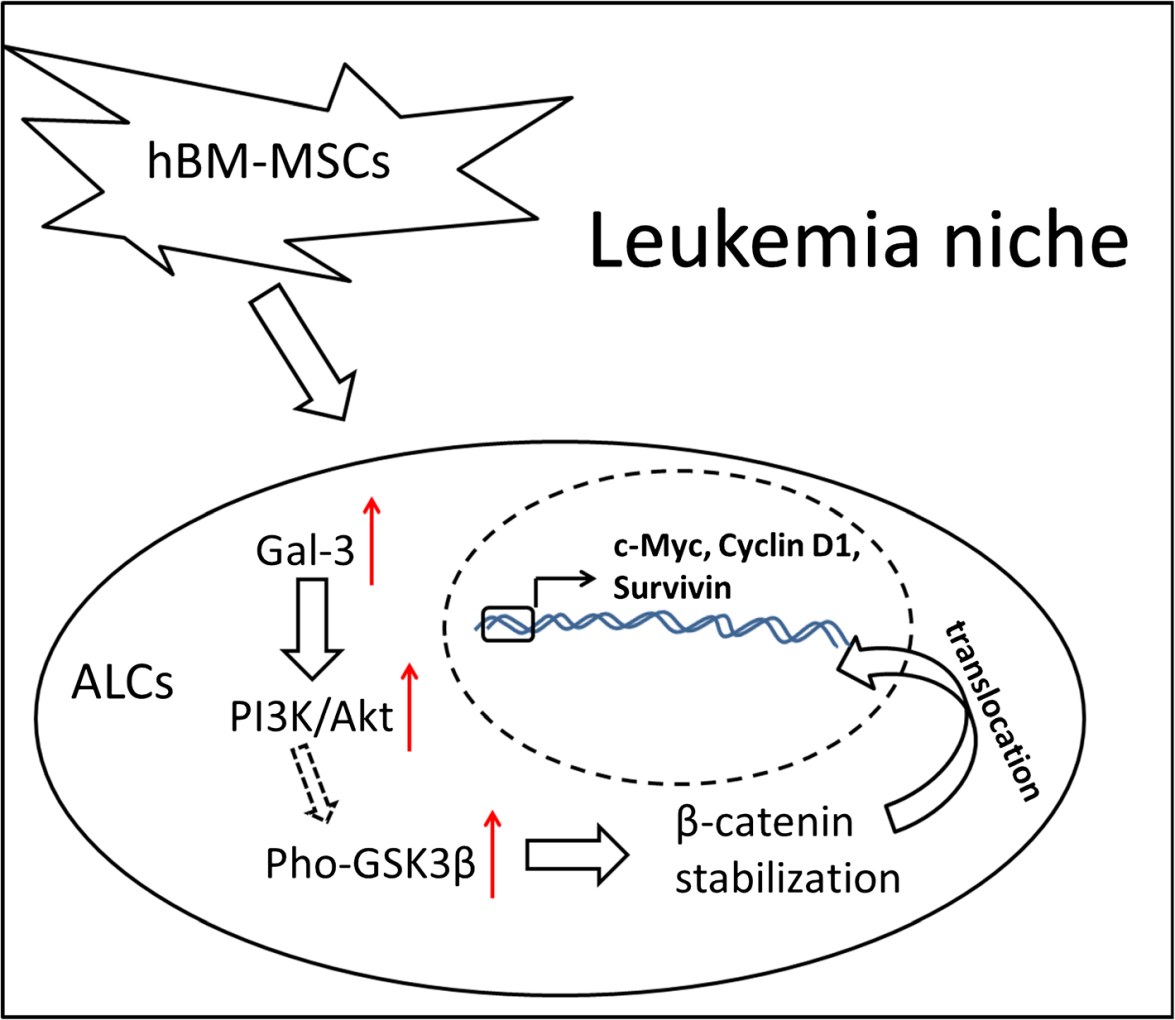
**Proposed model in which gal-3 mediates Wnt/β-catenin signaling in the BMM-induced drug resistance of AL.** hBM-MSCs induce gal-3 expression in ALCs. Gal-3 mediates Akt phosphorylation, which promotes phosphorylation of GSK-3β and thus decreases its activity. Inactivation of GSK-3β leads to a reduction in β-catenin degradation. Stabilized β-catenin translocates to the nucleus and activates transcription of its specific target genes, which ultimately leads to drug resistance in ALCs.

Collectively, we propose a model (Figure 6) in which hBM-MSCs induce drug resistance of ALCs by up-regulation of gal-3 expression. Gal-3 overexpression reduces β-catenin degradation through the PI3K/Akt pathway and phosphorylation of GSK-3β. Thus β-catenin is stabilized and translocated to the nucleus, where it activates the transcription of its downstream targets, ultimately leading to drug resistance of ALCs. Thus our study demonstrates a novel and possibly clinically-significant role of gal-3 in the bone marrow microenvironment of acute leukemia.

## Methods

### Cells and drugs

Human acute leukemia cell lines Reh (non-B non-T ALL) [44], Sup-B15 (B-ALL) [45], Jurkat (T-ALL) [46], and Kasumi-1 (AML, FAB M2) [47] were obtained from the Cell Bank of the Shanghai Institute of Biochemistry and Cell Biology, Chinese Academy of Sciences. Bone marrow was collected from healthy adult donors after they had provided informed consent, and the hBM-MSCs obtained were cultured in DMEM-low glucose (Gibco/Life Technologies, Carlsbad, CA, USA) supplemented with 10% fetal bovine serum (FBS) (Gibco), identified as described in our previous report [48]. Bone marrow mononuclear cells, of which more than 90% were malignant cells, from primary and refractory/relapsed acute leukemia (non-M3 AML and ALL) patients were purified by Ficoll-Paque isodensity gradient centrifugation (Tianjing, China). Idarubicin (IDA) and the Wnt signaling-specific inhibitor ICG-001 were obtained from Pfizer Inc., (New York, NY, USA) and from Selleck Chemicals, (Houston, TX, USA), respectively, and dissolved in phosphate-buffered saline (PBS) and dimethylsulfoxide (DMSO). Etoposide (VP-16) and the PI3K/Akt signaling inhibitor Ly294002 were purchased from Sigma-Aldrich (St Louis, MO, USA), and dissolved in DMSO. All of them were stored at −20°C.

### Cell culture and co-culture

Reh and Sup-B15 cells were maintained in IMDM (Gibco) supplemented with 10% FBS (Gibco), while Jurkat and Kasumi-1 cells were cultured in RPMI-1640 medium (Gibco) containing 10% FBS (Gibco). Human BM-MSCs at passage 3 to 7, displaying a homogeneous mesenchymal immunophenotype and multipotent differentiation potential, were used for co-culture experiments. They were seeded into 12- or 6-well plates at a density of 4 × 10^4^/mL. ALCs were added to the confluent hBM-MSC layer 1-2 days later at a ratio of 10:1. After ALCs adhered to the hBM-MSC layer, IDA or VP-16 was added. ICG-001 was added half an hour ahead of cytotoxic drugs. At indicated times, ALCs were collected, leaving the adherent stromal layer intact, and washed with PBS for subsequent analyses.

### Short hairpin RNA (shRNA) preparation and transfection

The shRNA against gal-3 in a lentiviral vector with green fluorescent protein, as well as the corresponding control vector were designed and synthesized by GenePharma Inc. (Shanghai, China). High-titer lentivirus was produced in 293 T cells by transfection of the lentiviral expression vector and packaging vectors, psPAX2 and pMD2.G (obtained from www.Addgene.org), using a calcium phosphate cell transfection kit according to the manufacturer’s instructions (Beyotime Institute of Biotechnology, Shanghai, China). The lentivirus was harvested 48 h later, filtered, enriched using 40% polyethylene glycol, and then used to infect acute leukemia cells. After transfection for 72 h, the efficiency was estimated by evaluation of EGFP expression by fluorescence microscopy and flow cytometry. The gal-3 specific shRNA sequence used in our study was 5′-GTACAATCATCGGGTTAAA-3′.

### CCK-8 assay for cell viability

Cell Counting Kit-8 (CCK-8) was obtained from Dojindo Laboratories (Kumamoto, Japan). Measurements were taken 48 h after drug exposure at the indicated concentrations. Absorbance was detected at 450/630 nm by a Benchmark microtiter plate reader (Bio-Rad Laboratories, Hercules, CA, USA). The relative cell viability was determined by (*A*_*co* − *cultured*_ − *A*_*medium*_)/(*A*_*cultured alone*_ − *A*_*medium*_) × 100 %.

### Cell apoptosis and cell cycle analyses

Acute leukemia cells cultured alone or co-cultured with hBM-MSCs were exposed to IDA or VP-16 at the indicated concentrations for 48 h, then cells were harvested, washed and resuspended in PBS. Apoptotic cells among non-transfected ALCs were identified by staining with Annexin V-FITC/PI (BD Pharmingen, Franklin Lakes, NJ, USA), while those in transfected ALCs were identified by staining with Annexin V-PE/7AAD (BD Pharmingen) according to the manufacturer’s instructions. The stained cells were analyzed by fluorescence activated cell sorting (FACS) (Beckman Coulter, Brea, CA, USA).

ALCs for cell cycle analyses were collected after 48 h of co-culture with hBM-MSCs. All cells were stained using the cell cycle detection kit (KeyGen Biotech. Co. Ltd., Nanjing, China) and analyzed by FACS. Cells in S and G_2_M phases were considered proliferative.

### Protein extraction and western blot analyses

Collected cells were lysed in lysis buffer containing 0.5 M Tris-HCl, pH 6.8, 2 mM EDTA, 10% glycerol, 2% SDS, 5% β-mercaptoethanol and protease inhibitors. Thirty to fifty micrograms of protein was separated by 10%-12% sodium dodecyl sulfate-polyacrylamide gel electrophoresis (SDS-PAGE) and electotransferred onto polyvinylidene fluoride membranes. The membranes were blocked in 5% bovine serum albumin (BSA) at room temperature for 2 h, incubated with primary antibodies overnight at 4°C, and then incubated with an IRDye secondary antibody (Li-Cor Biosciences, Lincoln, NE, USA) at room temperature for 1 h. The following antibodies were used: anti-gal-3 (Epitomics, Abcam, Cambridge, MA, USA), anti-β-catenin, anti-Akt, anti-pAkt (Cell Signaling Technology, Danvers, MA, USA), anti-pho-GSK-3β, anti-GSK-3β (Abcam). We also used anti-β-actin antibody (Sigma) as a control. Immunoreactive bands were visualized using an Odyssey infrared imaging system (Li-Cor). Signal intensity was quantified using Quantity One software (Bio-Rad) when necessary.

### RNA isolation and PCR analyses

Total RNA from collected cells was extracted using Trizol reagent (Takara Bio Inc., Shiga, Japan). One thousand nanograms of total RNA was used in a 2-step quantitative reverse transcription-PCR (Takara). Real time PCR was performed with the Roche Applied Science LightCycler 480 II Real-Time PCR System using the SYBR Green gene expression assay (Takara), according to the manufacturer’s instructions. The following primer sets were used (Sangon, Shanghai, China): Gal-3, 5′-GCCTTCCACTTTAACCCACG-3′ (forward) and 5′-AACCGACTGTCTTTCTTCCCTTC-3′ (reverse); β-catenin, 5′-CTGAGGACAAGCCACAAGATTA-3′ (forward) and 5′-ATCCACCAGAGTGAAAAGAACG-3′ (reverse); Cyclin D1, 5′-TCTACACCGACAACTCCATCC-3′ (forward) and 5′-GCATTTTGGAGAGGAAGTGTTC-3′ (reverse); c-Myc, 5′-CCTCCACTCGGAAGGACTATC-3′ (forward) and 5′-TGTTCGCCTCTTGACATTCTC-3′ (reverse); Survivin, 5′-CACCGCATCTCTACATTCAAGA -3′ (forward) and 5′-CAAGTCTGGCTCGTTCTCAGT-3′ (reverse); and GAPDH, 5′-AGAAGGCTGGGGCTCATTTG-3′ (forward) and 5′-AGGGGCCATCCACAGTCTTC-3′ (reverse). Independent triplicate samples were used in our experiments.

### Statistical analyses

Statistical analyses were performed using GraphPad Prism for Windows version 5.00 (GraphPad Software, San Diego, CA, USA) and SPSS 20.0. All data were presented as mean ± SD and statistical differences were evaluated using Student’s 2-tailed *t*-test (paired or unpaired, as appropriate) and Mann-Whitney *U* test (for data from AL patients). Differences were considered statistically significant at *P* < 0.05.

## Abbreviations

AL: Acute leukemia
MRD: Minimal residual disease
BMM: bone marrow microenvironment
ALCs: Acute leukemia cells
gal-3: Galectin-3
hBM-MSCs: Human bone marrow-derived mesenchymal stromal cells
GSK-3β: Glycogen synthase kinase
AML: Acute myeloid leukemia
ALL: Acute lymphoblastic leukemia
CML: Chronic myelogenous leukemia
FBS: fetal bovine serum
IDA: Idarubicin
VP-16: Etoposide
PBS: Phosphate-buffered saline
DMSO: Dimethylsulfoxide
CCK-8: Cell Counting Kit-8
FACS: Fluorescence activating cell sorter
BSA: Bull serum albumin

## References

[1] Pui CH, Evans WE (2006). Treatment of acute lymphoblastic leukemia. N Engl J Med.

[2] Zhao Y, Huang H, Wei G (2013). Novel agents and biomarkers for acute lymphoid leukemia. J Hematol Oncol.

[3] Patel JP, Gonen M, Figueroa ME, Fernandez H, Sun Z, Racevskis J (2012). Prognostic relevance of integrated genetic profiling in acute myeloid leukemia. N Engl J Med.

[4] Estey EH (2013). Acute myeloid leukemia: 2013 update on risk-stratification and management. Am J Hematol.

[5] Kern W, Danhauser-Riedl S, Ratei R, Schnittger S, Schoch C, Kolb HJ (2003). Detection of minimal residual disease in unselected patients with acute myeloid leukemia using multiparameter flow cytometry for definition of leukemia-associated immunophenotypes and determination of their frequencies in normal bone marrow. Haematologica.

[6] Ladetto M, Bruggemann M, Monitillo L, Ferrero S, Pepin F, Drandi D (2014). Next-generation sequencing and real-time quantitative PCR for minimal residual disease detection in B-cell disorders. Leukemia.

[7] Nagafuji K, Miyamoto T, Eto T, Kamimura T, Taniguchi S, Okamura T (2013). Monitoring of minimal residual disease (MRD) is useful to predict prognosis of adult patients with Ph-negative ALL: results of a prospective study (ALL MRD2002 Study). J Hematol Oncol.

[8] Azizidoost S, Babashah S, Rahim F, Shahjahani M, Saki N (2014). Bone marrow neoplastic niche in leukemia. Hematology.

[9] Malfuson JV, Boutin L, Clay D, Thepenier C, Desterke C, Torossian F (2014). SP/drug efflux functionality of hematopoietic progenitors is controlled by mesenchymal niche through VLA-4/CD44 axis. Leukemia.

[10] Burger JA, Kipps TJ (2006). CXCR4: a key receptor in the crosstalk between tumor cells and their microenvironment. Blood.

[11] Nwabo Kamdje AH, Mosna F, Bifari F, Lisi V, Bassi G, Malpeli G (2011). Notch-3 and Notch-4 signaling rescue from apoptosis human B-ALL cells in contact with human bone marrow-derived mesenchymal stromal cells. Blood.

[12] Sun Z, Wang S, Zhao RC (2014). The roles of mesenchymal stem cells in tumor inflammatory microenvironment. J Hematol Oncol.

[13] Pittenger MF, Mackay AM, Beck SC, Jaiswal RK, Douglas R, Mosca JD (1999). Multilineage potential of adult human mesenchymal stem cells. Science.

[14] Dumic J, Dabelic S, Flogel M (2006). Galectin-3: an open-ended story. Biochim Biophys Acta.

[15] Newlaczyl AU, Yu LG (2011). Galectin-3-A jack-of-all-trades in cancer. Cancer Lett.

[16] Yamamoto-Sugitani M, Kuroda J, Ashihara E, Nagoshi H, Kobayashi T, Matsumoto Y (2011). Galectin-3 (Gal-3) induced by leukemia microenvironment promotes drug resistance and bone marrow lodgment in chronic myelogenous leukemia. Proc Natl Acad Sci U S A.

[17] Fei F, Abdel-Azim H, Lim M, Arutyunyan A, von Itzstein M, Groffen J (2013). Galectin-3 in pre-B acute lymphoblastic leukemia. Leukemia.

[18] Cheng CL, Hou HA, Lee MC, Liu CY, Jhuang JY, Lai YJ (2013). Higher bone marrow LGALS3 expression is an independent unfavorable prognostic factor for overall survival in patients with acute myeloid leukemia. Blood.

[19] Song S, Mazurek N, Liu C, Sun Y, Ding QQ, Liu K (2009). Galectin-3 mediates nuclear beta-catenin accumulation and Wnt signaling in human colon cancer cells by regulation of glycogen synthase kinase-3beta activity. Cancer Res.

[20] Kobayashi T, Shimura T, Yajima T, Kubo N, Araki K, Tsutsumi S (2011). Transient gene silencing of galectin-3 suppresses pancreatic cancer cell migration and invasion through degradation of beta-catenin. Int J Cancer.

[21] Ge X, Wang X (2010). Role of Wnt canonical pathway in hematological malignancies. J Hematol Oncol.

[22] Staal F. Wnt Signaling Strength Regulates Normal Hematopoiesis and Its Deregulation Is Involved in Leukemia Development. Exp Hematol. 2012;40(8):S41–1. 10.1038/leu.2011.387 PMC3378318 22173215

[23] Luis TC, Ichii M, Brugman MH, Kincade P, Staal FJT (2012). Wnt signaling strength regulates normal hematopoiesis and its deregulation is involved in leukemia development. Leukemia.

[24] Yang Y, Mallampati S, Sun BH, Zhang J, Kim SB, Lee JS (2013). Wnt pathway contributes to the protection by bone marrow stromal cells of acute lymphoblastic leukemia cells and is a potential therapeutic target. Cancer Lett.

[25] He TC, Sparks AB, Rago C, Hermeking H, Zawel L, da Costa LT (1998). Identification of c-MYC as a target of the APC pathway. Science.

[26] Shtutman M, Zhurinsky J, Simcha I, Albanese C, D'Amico M, Pestell R (1999). The cyclin D1 gene is a target of the beta-catenin/LEF-1 pathway. Proc Natl Acad Sci U S A.

[27] Minke KS, Staib P, Puetter A, Gehrke I, Gandhirajan RK, Schlosser A (2009). Small molecule inhibitors of WNT signaling effectively induce apoptosis in acute myeloid leukemia cells. Eur J Haematol.

[28] Koh TJ, Bulitta CJ, Fleming JV, Dockray GJ, Varro A, Wang TC (2000). Gastrin is a target of the beta-catenin/TCF-4 growth-signaling pathway in a model of intestinal polyposis. J Clin Investig.

[29] Crawford HC, Fingleton BM, Rudolph-Owen LA, Goss KJH, Rubinfeld B, Polakis P (1999). The metalloproteinase matrilysin is a target of beta-catenin transactivation in intestinal tumors. Oncogene.

[30] Araki Y, Okamura S, Hussain SP, Nagashima M, He PJ, Shiseki M (2003). Regulation of cyclooxygenase-2 expression by the Wnt and ras pathways. Cancer Res.

[31] Lin HM, Pestell RG, Raz A, Kim HRC (2002). Galectin-3 enhances cyclin D-1 promoter activity through SP1 and a cAMP-responsive element in human breast epithelial cells. Oncogene.

[32] Wang YZ, Krivtsov AV, Sinha AU, North TE, Goessling W, Feng ZH (2010). The Wnt/beta-Catenin Pathway Is Required for the Development of Leukemia Stem Cells in AML. Science.

[33] Morgan RG, Ridsdale J, Tonks A, Darley RL (2014). Factors affecting the nuclear localization of beta-catenin in normal and malignant tissue. J Cell Biochem.

[34] Yamaguchi K, Lee SH, Eling TE, Baek SJ (2004). Identification of nonsteroidal anti-inflammatory drug-activated gene (NAG-1) as a novel downstream target of phosphatidylinositol 3-kinase/AKT/GSK-3beta pathway. J Biol Chem.

[35] Somervaille TC, Linch DC, Khwaja A (2001). Growth factor withdrawal from primary human erythroid progenitors induces apoptosis through a pathway involving glycogen synthase kinase-3 and Bax. Blood.

[36] Liu J, Han G, Liu H, Qin C (2013). Suppression of cholangiocarcinoma cell growth by human umbilical cord mesenchymal stem cells: a possible role of Wnt and Akt signaling. PLoS One.

[37] Chauhan D, Li G, Podar K, Hideshima T, Neri P, He D (2005). A novel carbohydrate-based therapeutic GCS-100 overcomes bortezomib resistance and enhances dexamethasone-induced apoptosis in multiple myeloma cells. Cancer Res.

[38] Streetly MJ, Maharaj L, Joel S, Schey SA, Gribben JG, Cotter FE (2010). GCS-100, a novel galectin-3 antagonist, modulates MCL-1, NOXA, and cell cycle to induce myeloma cell death. Blood.

[39] O'Brien S, Kay NE (2011). Maintenance therapy for B-chronic lymphocytic leukemia. Clin Adv Hematol Oncol.

[40] Clark MC, Pang M, Hsu DK, Liu FT, de Vos S, Gascoyne RD (2012). Galectin-3 binds to CD45 on diffuse large B-cell lymphoma cells to regulate susceptibility to cell death. Blood.

[41] Liu GY, Xu Y, Li Y, Wang LH, Liu YJ, Zhu D (2013). Secreted galectin-3 as a possible biomarker for the immunomodulatory potential of human umbilical cord mesenchymal stromal cells. Cytotherapy.

[42] Gutierrez SE, Romero-Oliva FA (2013). Epigenetic changes: a common theme in acute myelogenous leukemogenesis. J Hematol Oncol.

[43] Ramasamy S, Duraisamy S, Barbashov S, Kawano T, Kharbanda S, Kufe D (2007). The MUC1 and galectin-3 oncoproteins function in a microRNA-dependent regulatory loop. Mol Cell.

[44] Rosenfeld C, Goutner A, Venuat AM, Choquet C, Pico JL, Dore JF (1977). An effect human leukaemic cell line: Reh. Eur J Cancer.

[45] Clark SS, McLaughlin J, Timmons M, Pendergast AM, Ben-Neriah Y, Dow LW (1988). Expression of a distinctive BCR-ABL oncogene in Ph1-positive acute lymphocytic leukemia (ALL). Science.

[46] Gillis S, Watson J (1980). Biochemical and biological characterization of lymphocyte regulatory molecules. V. Identification of an interleukin 2-producing human leukemia T cell line. J Exp Med.

[47] Asou H, Tashiro S, Hamamoto K, Otsuji A, Kita K, Kamada N (1991). Establishment of a human acute myeloid leukemia cell line (Kasumi-1) with 8;21 chromosome translocation. Blood.

[48] Zhao YM, Li JY, Lan JP, Lai XY, Luo Y, Sun J (2008). Cell cycle dependent telomere regulation by telomerase in human bone marrow mesenchymal stem cells. Biochem Biophys Res Commun.

